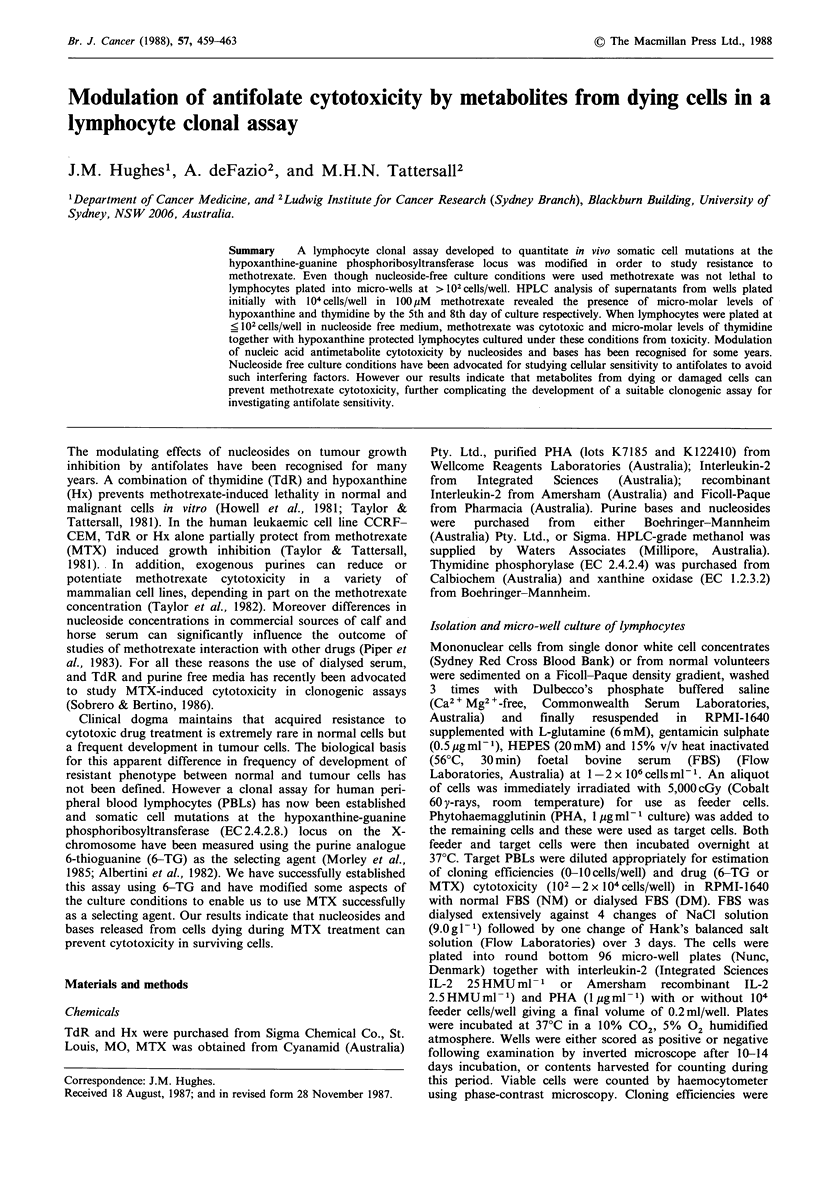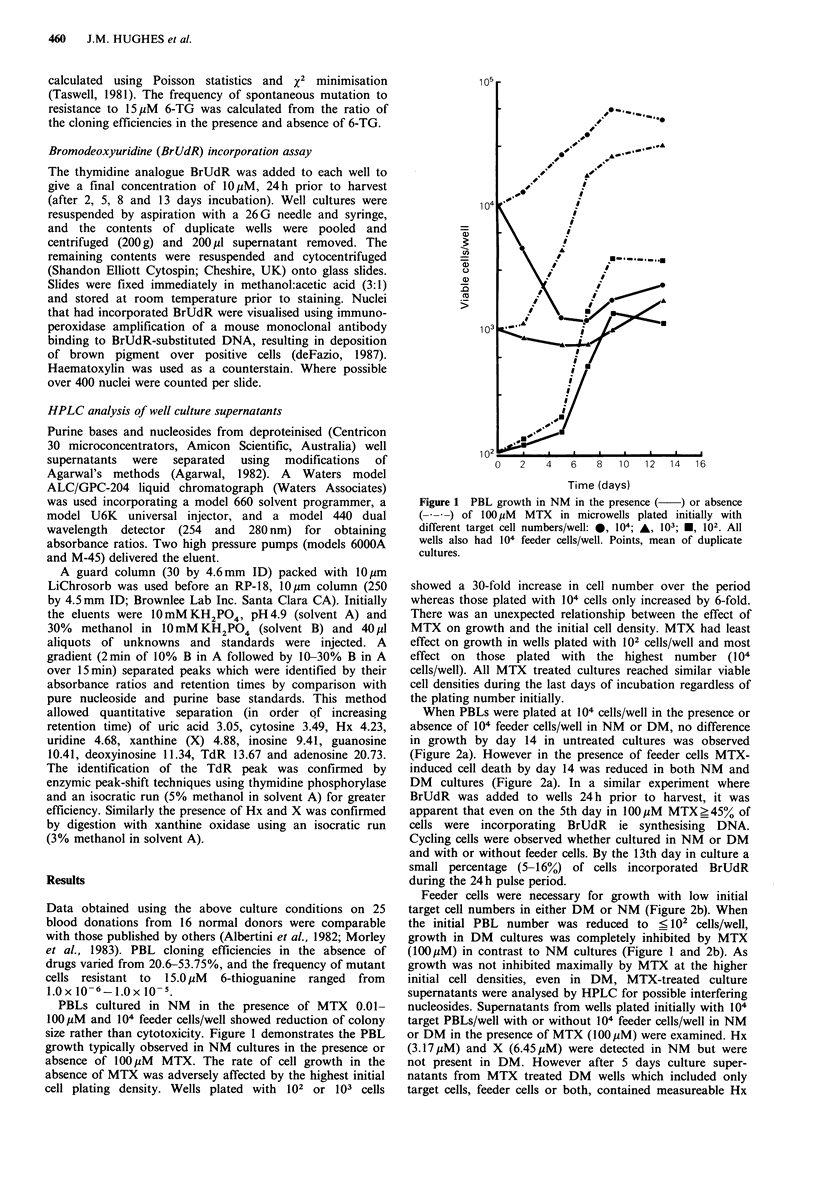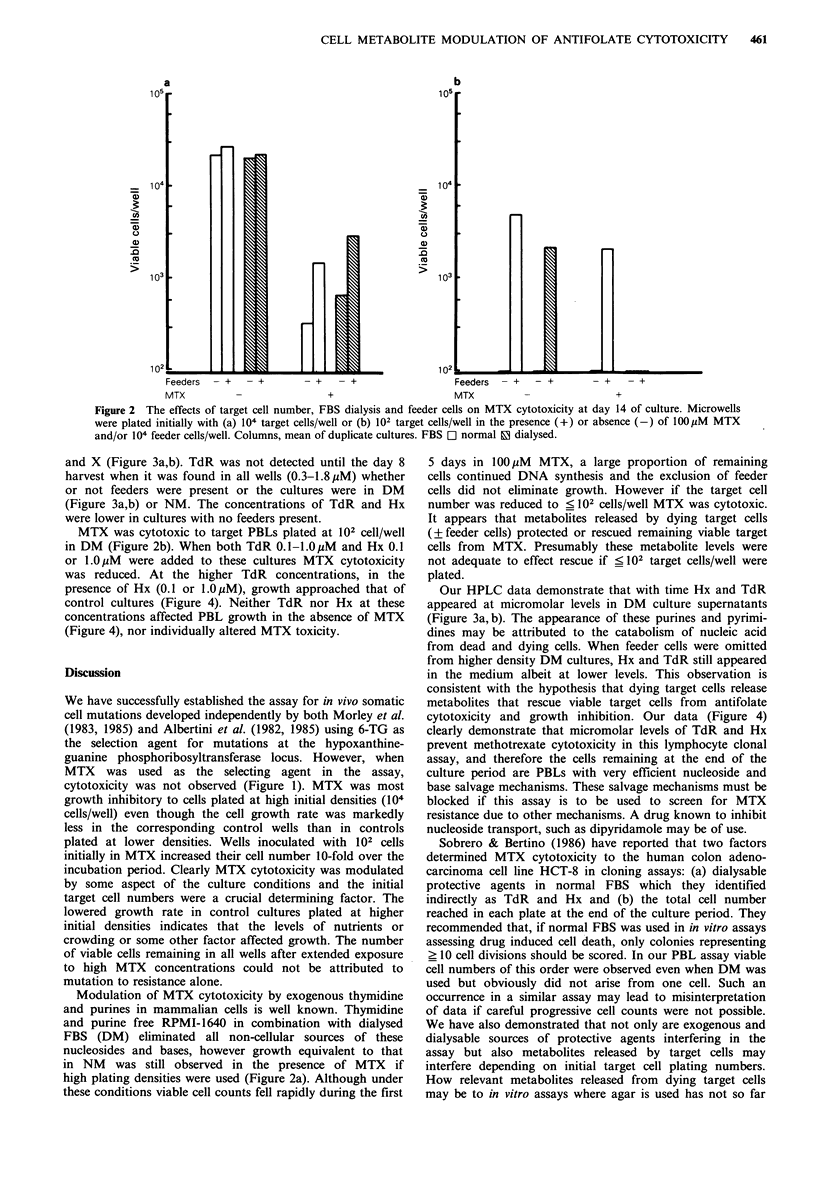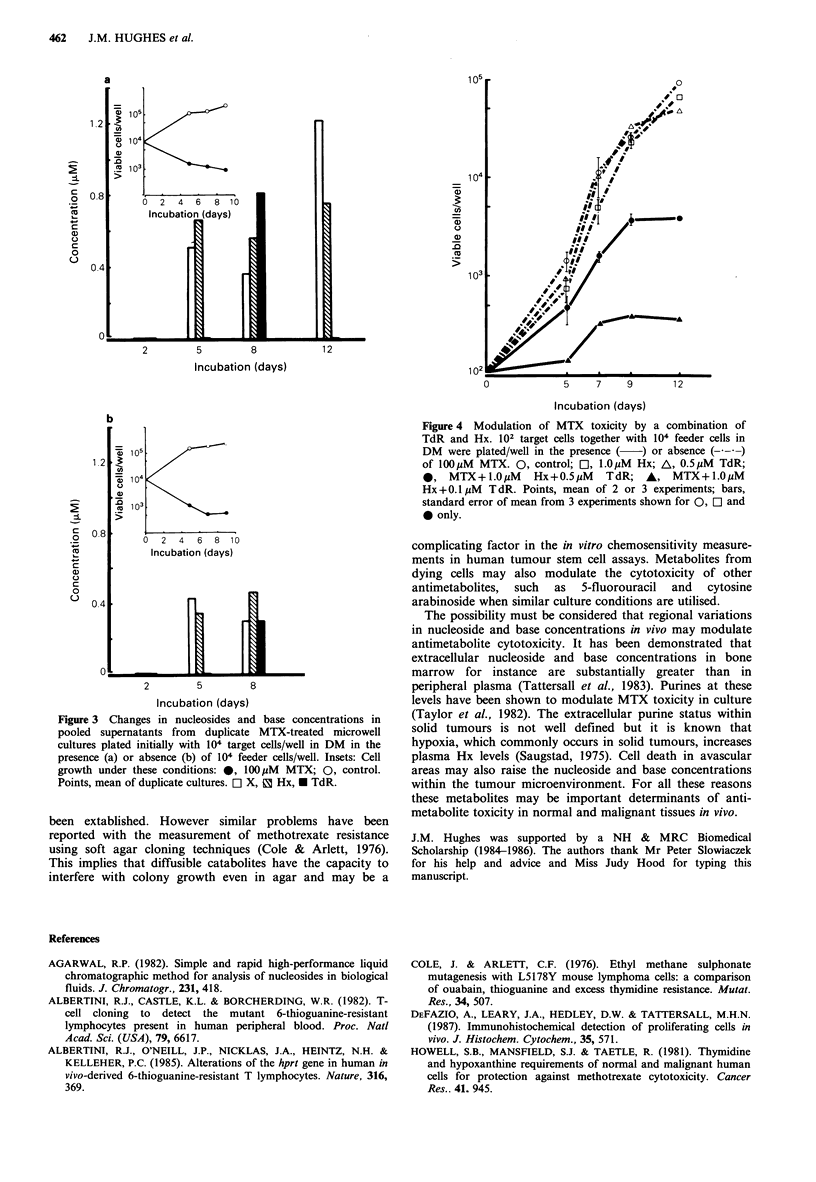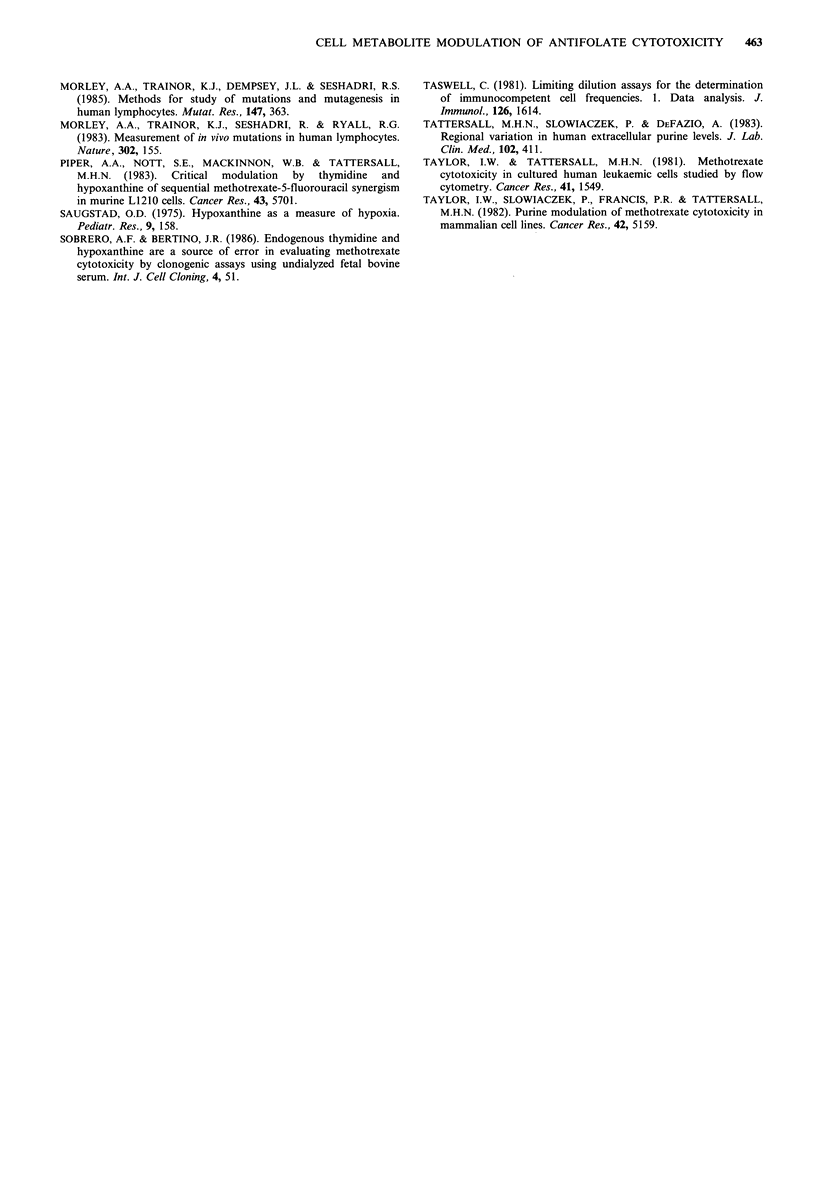# Modulation of antifolate cytotoxicity by metabolites from dying cells in a lymphocyte clonal assay.

**DOI:** 10.1038/bjc.1988.107

**Published:** 1988-05

**Authors:** J. M. Hughes, A. deFazio, M. H. Tattersall

**Affiliations:** Department of Cancer Medicine, University of Sydney, NSW, Australia.

## Abstract

A lymphocyte clonal assay developed to quantitate in vivo somatic cell mutations at the hypoxanthine-guanine phosphoribosyltransferase locus was modified in order to study resistance to methotrexate. Even though nucleoside-free culture conditions were used methotrexate was not lethal to lymphocytes plated into micro-wells at greater than 10(2) cells/well. HPLC analysis of supernatants from wells plated initially with 10(4) cells/well in 100 microM methotrexate revealed the presence of micro-molar levels of hypoxanthine and thymidine by the 5th and 8th day of culture respectively. When lymphocytes were plated at less than or equal to 10(2) cells/well in nucleoside free medium, methotrexate was cytotoxic and micro-molar levels of thymidine together with hypoxanthine protected lymphocytes cultured under these conditions from toxicity. Modulation of nucleic acid antimetabolite cytotoxicity by nucleosides and bases has been recognised for some years. Nucleoside free culture conditions have been advocated for studying cellular sensitivity to antifolates to avoid such interfering factors. However our results indicate that metabolites from dying or damaged cells can prevent methotrexate cytotoxicity, further complicating the development of a suitable clonogenic assay for investigating antifolate sensitivity.


Br. J. Cancer (1988), 57, 459–463 © The Macmillan Press Ltd., 1988

### Modulation of antifolate cytotoxicity by metabolites from dying cells in a lymphocyte clonal assay

J.M. Hughes[1], A. deFazio[2], and M.H.N. Tattersall[2]

[1]*Department of Cancer Medicine, and* [2]*Ludwig Institute for Cancer Research (Sydney Branch), Blackburn Building, University of Sydney, NSW 2006, Australia.*

**Summary** A lymphocyte clonal assay developed to quantitate *in vivo* somatic cell mutations at the hypoxanthine-guanine phosphoribosyltransferase locus was modified in order to study resistance to methotrexate. Even though nucleoside-free culture conditions were used methotrexate was not lethal to lymphocytes plated into micro-wells at $>10^2$ cells/well. HPLC analysis of supernatants from wells plated initially with $10^4$ cells/well in 100 $\mu$M methotrexate revealed the presence of micro-molar levels of hypoxanthine and thymidine by the 5th and 8th day of culture respectively. When lymphocytes were plated at $\leq 10^2$ cells/well in nucleoside free medium, methotrexate was cytotoxic and micro-molar levels of thymidine together with hypoxanthine protected lymphocytes cultured under these conditions from toxicity. Modulation of nucleic acid antimetabolite cytotoxicity by nucleosides and bases has been recognised for some years. Nucleoside free culture conditions have been advocated for studying cellular sensitivity to antifolates to avoid such interfering factors. However our results indicate that metabolites from dying or damaged cells can prevent methotrexate cytotoxicity, further complicating the development of a suitable clonogenic assay for investigating antifolate sensitivity.

The modulating effects of nucleosides on tumour growth inhibition by antifolates have been recognised for many years. A combination of thymidine (TdR) and hypoxanthine (Hx) prevents methotrexate-induced lethality in normal and malignant cells *in vitro* (Howell *et al.*, 1981; Taylor & Tattersall, 1981). In the human leukaemic cell line CCRF-CEM, TdR or Hx alone partially protect from methotrexate (MTX) induced growth inhibition (Taylor & Tattersall, 1981). In addition, exogenous purines can reduce or potentiate methotrexate cytotoxicity in a variety of mammalian cell lines, depending in part on the methotrexate concentration (Taylor *et al.*, 1982). Moreover differences in nucleoside concentrations in commercial sources of calf and horse serum can significantly influence the outcome of studies of methotrexate interaction with other drugs (Piper *et al.*, 1983). For all these reasons the use of dialysed serum, and TdR and purine free media has recently been advocated to study MTX-induced cytotoxicity in clonogenic assays (Sobrero & Bertino, 1986).

Clinical dogma maintains that acquired resistance to cytotoxic drug treatment is extremely rare in normal cells but a frequent development in tumour cells. The biological basis for this apparent difference in frequency of development of resistant phenotype between normal and tumour cells has not been defined. However a clonal assay for human peripheral blood lymphocytes (PBLs) has now been established and somatic cell mutations at the hypoxanthine-guanine phosphoribosyltransferase (EC 2.4.2.8.) locus on the X-chromosome have been measured using the purine analogue 6-thioguanine (6–TG) as the selecting agent (Morley *et al.*, 1985; Albertini *et al.*, 1982). We have successfully established this assay using 6–TG and have modified some aspects of the culture conditions to enable us to use MTX successfully as a selecting agent. Our results indicate that nucleosides and bases released from cells dying during MTX treatment can prevent cytotoxicity in surviving cells.

### Materials and methods

### *Chemicals*

TdR and Hx were purchased from Sigma Chemical Co., St. Louis, MO, MTX was obtained from Cyanamid (Australia) Pty. Ltd., purified PHA (lots K7185 and K122410) from Wellcome Reagents Laboratories (Australia); Interleukin-2 from Integrated Sciences (Australia); recombinant Interleukin-2 from Amersham (Australia) and Ficoll-Paque from Pharmacia (Australia). Purine bases and nucleosides were purchased from either Boehringer–Mannheim (Australia) Pty. Ltd., or Sigma. HPLC-grade methanol was supplied by Waters Associates (Millipore, Australia). Thymidine phosphorylase (EC 2.4.2.4) was purchased from Calbiochem (Australia) and xanthine oxidase (EC 1.2.3.2) from Boehringer–Mannheim.

### *Isolation and micro-well culture of lymphocytes*

Mononuclear cells from single donor white cell concentrates (Sydney Red Cross Blood Bank) or from normal volunteers were sedimented on a Ficoll–Paque density gradient, washed 3 times with Dulbecco's phosphate buffered saline ($Ca^{2+}$ $Mg^{2+}$-free, Commonwealth Serum Laboratories, Australia) and finally resuspended in RPMI-1640 supplemented with L-glutamine (6 mM), gentamicin sulphate (0.5 $\mu$g ml$^{-1}$), HEPES (20 mM) and 15% v/v heat inactivated (56°C, 30 min) foetal bovine serum (FBS) (Flow Laboratories, Australia) at $1-2\times10^6$ cells ml$^{-1}$. An aliquot of cells was immediately irradiated with 5,000 cGy (Cobalt 60 $\gamma$-rays, room temperature) for use as feeder cells. Phytohaemagglutinin (PHA, 1 $\mu$g ml$^{-1}$ culture) was added to the remaining cells and these were used as target cells. Both feeder and target cells were then incubated overnight at 37°C. Target PBLs were diluted appropriately for estimation of cloning efficiencies (0–10 cells/well) and drug (6–TG or MTX) cytotoxicity ($10^2-2\times10^4$ cells/well) in RPMI-1640 with normal FBS (NM) or dialysed FBS (DM). FBS was dialysed extensively against 4 changes of NaCl solution (9.0 g l$^{-1}$) followed by one change of Hank's balanced salt solution (Flow Laboratories) over 3 days. The cells were plated into round bottom 96 micro-well plates (Nunc, Denmark) together with interleukin-2 (Integrated Sciences IL-2 25 HMU ml$^{-1}$ or Amersham recombinant IL-2 2.5 HMU ml$^{-1}$) and PHA (1 $\mu$g ml$^{-1}$) with or without $10^4$ feeder cells/well giving a final volume of 0.2 ml/well. Plates were incubated at 37°C in a 10% $CO_2$, 5% $O_2$ humidified atmosphere. Wells were either scored as positive or negative following examination by inverted microscope after 10–14 days incubation, or contents harvested for counting during this period. Viable cells were counted by haemocytometer using phase-contrast microscopy. Cloning efficiencies were

Correspondence: J.M. Hughes.
Received 18 August, 1987; and in revised form 28 November 1987.

calculated using Poisson statistics and $\chi^2$ minimisation (Taswell, 1981). The frequency of spontaneous mutation to resistance to 15 $\mu$M 6-TG was calculated from the ratio of the cloning efficiencies in the presence and absence of 6-TG.

*Bromodeoxyuridine (BrUdR) incorporation assay*

The thymidine analogue BrUdR was added to each well to give a final concentration of 10 $\mu$M, 24 h prior to harvest (after 2, 5, 8 and 13 days incubation). Well cultures were resuspended by aspiration with a 26 G needle and syringe, and the contents of duplicate wells were pooled and centrifuged (200 g) and 200 $\mu$l supernatant removed. The remaining contents were resuspended and cytocentrifuged (Shandon Elliott Cytospin; Cheshire, UK) onto glass slides. Slides were fixed immediately in methanol:acetic acid (3:1) and stored at room temperature prior to staining. Nuclei that had incorporated BrUdR were visualised using immunoperoxidase amplification of a mouse monoclonal antibody binding to BrUdR-substituted DNA, resulting in deposition of brown pigment over positive cells (deFazio, 1987). Haematoxylin was used as a counterstain. Where possible over 400 nuclei were counted per slide.

*HPLC analysis of well culture supernatants*

Purine bases and nucleosides from deproteinised (Centricon 30 microconcentrators, Amicon Scientific, Australia) well supernatants were separated using modifications of Agarwal's methods (Agarwal, 1982). A Waters model ALC/GPC-204 liquid chromatograph (Waters Associates) was used incorporating a model 660 solvent programmer, a model U6K universal injector, and a model 440 dual wavelength detector (254 and 280 nm) for obtaining absorbance ratios. Two high pressure pumps (models 6000A and M-45) delivered the eluent.

A guard column (30 by 4.6 mm ID) packed with 10 $\mu$m LiChrosorb was used before an RP-18, 10 $\mu$m column (250 by 4.5 mm ID; Brownlee Lab Inc. Santa Clara CA). Initially the eluents were 10 mM $KH_2PO_4$, pH 4.9 (solvent A) and 30% methanol in 10 mM $KH_2PO_4$ (solvent B) and 40 $\mu$l aliquots of unknowns and standards were injected. A gradient (2 min of 10% B in A followed by 10–30% B in A over 15 min) separated peaks which were identified by their absorbance ratios and retention times by comparison with pure nucleoside and purine base standards. This method allowed quantitative separation (in order of increasing retention time) of uric acid 3.05, cytosine 3.49, Hx 4.23, uridine 4.68, xanthine (X) 4.88, inosine 9.41, guanosine 10.41, deoxyinosine 11.34, TdR 13.67 and adenosine 20.73. The identification of the TdR peak was confirmed by enzymic peak-shift techniques using thymidine phosphorylase and an isocratic run (5% methanol in solvent A) for greater efficiency. Similarly the presence of Hx and X was confirmed by digestion with xanthine oxidase using an isocratic run (3% methanol in solvent A).

### Results

Data obtained using the above culture conditions on 25 blood donations from 16 normal donors were comparable with those published by others (Albertini et al., 1982; Morley et al., 1983). PBL cloning efficiencies in the absence of drugs varied from 20.6–53.75%, and the frequency of mutant cells resistant to 15.0 $\mu$M 6-thioguanine ranged from $1.0 \times 10^{-6} - 1.0 \times 10^{-5}$.

PBLs cultured in NM in the presence of MTX 0.01–100 $\mu$M and $10^4$ feeder cells/well showed reduction of colony size rather than cytotoxicity. Figure 1 demonstrates the PBL growth typically observed in NM cultures in the presence or absence of 100 $\mu$M MTX. The rate of cell growth in the absence of MTX was adversely affected by the highest initial cell plating density. Wells plated with $10^2$ or $10^3$ cells

**Figure 1** PBL growth in NM in the presence (——) or absence (–·–·–) of 100 $\mu$M MTX in microwells plated initially with different target cell numbers/well: ●, $10^4$; ▲, $10^3$; ■, $10^2$. All wells also had $10^4$ feeder cells/well. Points, mean of duplicate cultures.

showed a 30-fold increase in cell number over the period whereas those plated with $10^4$ cells only increased by 6-fold. There was an unexpected relationship between the effect of MTX on growth and the initial cell density. MTX had least effect on growth in wells plated with $10^2$ cells/well and most effect on those plated with the highest number ($10^4$ cells/well). All MTX treated cultures reached similar viable cell densities during the last days of incubation regardless of the plating number initially.

When PBLs were plated at $10^4$ cells/well in the presence or absence of $10^4$ feeder cells/well in NM or DM, no difference in growth by day 14 in untreated cultures was observed (Figure 2a). However in the presence of feeder cells MTX-induced cell death by day 14 was reduced in both NM and DM cultures (Figure 2a). In a similar experiment where BrUdR was added to wells 24 h prior to harvest, it was apparent that even on the 5th day in 100 $\mu$M MTX $\geq$ 45% of cells were incorporating BrUdR ie synthesising DNA. Cycling cells were observed whether cultured in NM or DM and with or without feeder cells. By the 13th day in culture a small percentage (5–16%) of cells incorporated BrUdR during the 24 h pulse period.

Feeder cells were necessary for growth with low initial target cell numbers in either DM or NM (Figure 2b). When the initial PBL number was reduced to $\leq 10^2$ cells/well, growth in DM cultures was completely inhibited by MTX (100 $\mu$M) in contrast to NM cultures (Figure 1 and 2b). As growth was not inhibited maximally by MTX at the higher initial cell densities, even in DM, MTX-treated culture supernatants were analysed by HPLC for possible interfering nucleosides. Supernatants from wells plated initially with $10^4$ target PBLs/well with or without $10^4$ feeder cells/well in NM or DM in the presence of MTX (100 $\mu$M) were examined. Hx (3.17 $\mu$M) and X (6.45 $\mu$M) were detected in NM but were not present in DM. However after 5 days culture supernatants from MTX treated DM wells which included only target cells, feeder cells or both, contained measureable Hx

**Figure 2** The effects of target cell number, FBS dialysis and feeder cells on MTX cytotoxicity at day 14 of culture. Microwells were plated initially with (a) $10^4$ target cells/well or (b) $10^2$ target cells/well in the presence (+) or absence (–) of 100 $\mu$M MTX and/or $10^4$ feeder cells/well. Columns, mean of duplicate cultures. FBS □ normal ▧ dialysed.

and X (Figure 3a,b). TdR was not detected until the day 8 harvest when it was found in all wells (0.3–1.8 $\mu$M) whether or not feeders were present or the cultures were in DM (Figure 3a,b) or NM. The concentrations of TdR and Hx were lower in cultures with no feeders present.

MTX was cytotoxic to target PBLs plated at $10^2$ cell/well in DM (Figure 2b). When both TdR 0.1–1.0 $\mu$M and Hx 0.1 or 1.0 $\mu$M were added to these cultures MTX cytotoxicity was reduced. At the higher TdR concentrations, in the presence of Hx (0.1 or 1.0 $\mu$M), growth approached that of control cultures (Figure 4). Neither TdR nor Hx at these concentrations affected PBL growth in the absence of MTX (Figure 4), nor individually altered MTX toxicity.

### Discussion

We have successfully established the assay for *in vivo* somatic cell mutations developed independently by both Morley *et al.* (1983, 1985) and Albertini *et al.* (1982, 1985) using 6-TG as the selection agent for mutations at the hypoxanthine-guanine phosphoribosyltransferase locus. However, when MTX was used as the selecting agent in the assay, cytotoxicity was not observed (Figure 1). MTX was most growth inhibitory to cells plated at high initial densities ($10^4$ cells/well) even though the cell growth rate was markedly less in the corresponding control wells than in controls plated at lower densities. Wells inoculated with $10^2$ cells initially in MTX increased their cell number 10-fold over the incubation period. Clearly MTX cytotoxicity was modulated by some aspect of the culture conditions and the initial target cell numbers were a crucial determining factor. The lowered growth rate in control cultures plated at higher initial densities indicates that the levels of nutrients or crowding or some other factor affected growth. The number of viable cells remaining in all wells after extended exposure to high MTX concentrations could not be attributed to mutation to resistance alone.

Modulation of MTX cytotoxicity by exogenous thymidine and purines in mammalian cells is well known. Thymidine and purine free RPMI-1640 in combination with dialysed FBS (DM) eliminated all non-cellular sources of these nucleosides and bases, however growth equivalent to that in NM was still observed in the presence of MTX if high plating densities were used (Figure 2a). Although under these conditions viable cell counts fell rapidly during the first 5 days in 100 $\mu$M MTX, a large proportion of remaining cells continued DNA synthesis and the exclusion of feeder cells did not eliminate growth. However if the target cell number was reduced to $\leq 10^2$ cells/well MTX was cytotoxic. It appears that metabolites released by dying target cells ($\pm$ feeder cells) protected or rescued remaining viable target cells from MTX. Presumably these metabolite levels were not adequate to effect rescue if $\leq 10^2$ target cells/well were plated.

Our HPLC data demonstrate that with time Hx and TdR appeared at micromolar levels in DM culture supernatants (Figure 3a, b). The appearance of these purines and pyrimidines may be attributed to the catabolism of nucleic acid from dead and dying cells. When feeder cells were omitted from higher density DM cultures, Hx and TdR still appeared in the medium albeit at lower levels. This observation is consistent with the hypothesis that dying target cells release metabolites that rescue viable target cells from antifolate cytotoxicity and growth inhibition. Our data (Figure 4) clearly demonstrate that micromolar levels of TdR and Hx prevent methotrexate cytotoxicity in this lymphocyte clonal assay, and therefore the cells remaining at the end of the culture period are PBLs with very efficient nucleoside and base salvage mechanisms. These salvage mechanisms must be blocked if this assay is to be used to screen for MTX resistance due to other mechanisms. A drug known to inhibit nucleoside transport, such as dipyridamole may be of use.

Sobrero & Bertino (1986) have reported that two factors determined MTX cytotoxicity to the human colon adenocarcinoma cell line HCT-8 in cloning assays: (a) dialysable protective agents in normal FBS which they identified indirectly as TdR and Hx and (b) the total cell number reached in each plate at the end of the culture period. They recommended that, if normal FBS was used in *in vitro* assays assessing drug induced cell death, only colonies representing $\geq 10$ cell divisions should be scored. In our PBL assay viable cell numbers of this order were observed even when DM was used but obviously did not arise from one cell. Such an occurrence in a similar assay may lead to misinterpretation of data if careful progressive cell counts were not possible. We have also demonstrated that not only are exogenous and dialysable sources of protective agents interfering in the assay but also metabolites released by target cells may interfere depending on initial target cell plating numbers. How relevant metabolites released from dying target cells may be to *in vitro* assays where agar is used has not so far

Figure 3 Changes in nucleosides and base concentrations in pooled supernatants from duplicate MTX-treated microwell cultures plated initially with $10^4$ target cells/well in DM in the presence (a) or absence (b) of $10^4$ feeder cells/well. Insets: Cell growth under these conditions: ●, 100 μM MTX; ○, control. Points, mean of duplicate cultures. □ X, ▧ Hx, ■ TdR.

Figure 4 Modulation of MTX toxicity by a combination of TdR and Hx. $10^2$ target cells together with $10^4$ feeder cells in DM were plated/well in the presence (——) or absence (–·–·–) of 100 μM MTX. ○, control; □, 1.0 μM Hx; △, 0.5 μM TdR; ●, MTX + 1.0 μM Hx + 0.5 μM TdR; ▲, MTX + 1.0 μM Hx + 0.1 μM TdR. Points, mean of 2 or 3 experiments; bars, standard error of mean from 3 experiments shown for ○, □ and ● only.

been extablished. However similar problems have been reported with the measurement of methotrexate resistance using soft agar cloning techniques (Cole & Arlett, 1976). This implies that diffusible catabolites have the capacity to interfere with colony growth even in agar and may be a complicating factor in the *in vitro* chemosensitivity measurements in human tumour stem cell assays. Metabolites from dying cells may also modulate the cytotoxicity of other antimetabolites, such as 5-fluorouracil and cytosine arabinoside when similar culture conditions are utilised.

The possibility must be considered that regional variations in nucleoside and base concentrations *in vivo* may modulate antimetabolite cytotoxicity. It has been demonstrated that extracellular nucleoside and base concentrations in bone marrow for instance are substantially greater than in peripheral plasma (Tattersall *et al.*, 1983). Purines at these levels have been shown to modulate MTX toxicity in culture (Taylor *et al.*, 1982). The extracellular purine status within solid tumours is not well defined but it is known that hypoxia, which commonly occurs in solid tumours, increases plasma Hx levels (Saugstad, 1975). Cell death in avascular areas may also raise the nucleoside and base concentrations within the tumour microenvironment. For all these reasons these metabolites may be important determinants of antimetabolite toxicity in normal and malignant tissues *in vivo*.

J.M. Hughes was supported by a NH & MRC Biomedical Scholarship (1984–1986). The authors thank Mr Peter Slowiaczek for his help and advice and Miss Judy Hood for typing this manuscript.

### References

AGARWAL, R.P. (1982). Simple and rapid high-performance liquid chromatographic method for analysis of nucleosides in biological fluids. *J. Chromatogr.*, **231**, 418.

ALBERTINI, R.J., CASTLE, K.L. & BORCHERDING, W.R. (1982). T-cell cloning to detect the mutant 6-thioguanine-resistant lymphocytes present in human peripheral blood. *Proc. Natl Acad. Sci.* (*USA*), **79**, 6617.

ALBERTINI, R.J., O'NEILL, J.P., NICKLAS, J.A., HEINTZ, N.H. & KELLEHER, P.C. (1985). Alterations of the *hprt* gene in human *in vivo*-derived 6-thioguanine-resistant T lymphocytes. *Nature*, **316**, 369.

COLE, J. & ARLETT, C.F. (1976). Ethyl methane sulphonate mutagenesis with L5178Y mouse lymphoma cells: a comparison of ouabain, thioguanine and excess thymidine resistance. *Mutat. Res.*, **34**, 507.

DEFAZIO, A., LEARY, J.A., HEDLEY, D.W. & TATTERSALL, M.H.N. (1987). Immunohistochemical detection of proliferating cells *in vivo*. *J. Histochem. Cytochem.*, **35**, 571.

HOWELL, S.B., MANSFIELD, S.J. & TAETLE, R. (1981). Thymidine and hypoxanthine requirements of normal and malignant human cells for protection against methotrexate cytotoxicity. *Cancer Res.*, **41**, 945.

MORLEY, A.A., TRAINOR, K.J., DEMPSEY, J.L. & SESHADRI, R.S. (1985). Methods for study of mutations and mutagenesis in human lymphocytes. *Mutat. Res.*, **147**, 363.

MORLEY, A.A., TRAINOR, K.J., SESHADRI, R. & RYALL, R.G. (1983). Measurement of *in vivo* mutations in human lymphocytes. *Nature*, **302**, 155.

PIPER, A.A., NOTT, S.E., MACKINNON, W.B. & TATTERSALL, M.H.N. (1983). Critical modulation by thymidine and hypoxanthine of sequential methotrexate-5-fluorouracil synergism in murine L1210 cells. *Cancer Res.*, **43**, 5701.

SAUGSTAD, O.D. (1975). Hypoxanthine as a measure of hypoxia. *Pediatr. Res.*, **9**, 158.

SOBRERO, A.F. & BERTINO, J.R. (1986). Endogenous thymidine and hypoxanthine are a source of error in evaluating methotrexate cytotoxicity by clonogenic assays using undialyzed fetal bovine serum. *Int. J. Cell Cloning*, **4**, 51.

TASWELL, C. (1981). Limiting dilution assays for the determination of immunocompetent cell frequencies. 1. Data analysis. *J. Immunol.*, **126**, 1614.

TATTERSALL, M.H.N., SLOWIACZEK, P. & DEFAZIO, A. (1983). Regional variation in human extracellular purine levels. *J. Lab. Clin. Med.*, **102**, 411.

TAYLOR, I.W. & TATTERSALL, M.H.N. (1981). Methotrexate cytotoxicity in cultured human leukaemic cells studied by flow cytometry. *Cancer Res.*, **41**, 1549.

TAYLOR, I.W., SLOWIACZEK, P., FRANCIS, P.R. & TATTERSALL, M.H.N. (1982). Purine modulation of methotrexate cytotoxicity in mammalian cell lines. *Cancer Res.*, **42**, 5159.